# Abnormal “*Escherichia Shigella*-EAT-LAAD” axis in HFrEF patients with AF and the regulatory effect of SGLT2 inhibitors

**DOI:** 10.3389/fcvm.2026.1872678

**Published:** 2026-08-21

**Authors:** Jia-Ming Han, Fang-Yi Zhu, Xiao-Hu Niu, Jian-Feng Hu, Shi-Ming Fan, Zuo-Hua Ying, Yu-Lan Ma

**Affiliations:** 1 Qinghai University, Xining, China; 2 Qinghai Cardio-Cerebrovascular Specialty Hospital, Xining, China; 3 Department of Cardiology, Affiliated Hospital of Qinghai University, Xining, China

**Keywords:** AF, epicardial adipose tissue, gut microbiota, HFREF, SGLT2I

## Abstract

**Background:**

The role of epicardial adipose tissue (EAT) and gut microbiota (GM) in heart failure (HF) and atrial fibrillation (AF) have been extensively researched, but the role of them in individuals with concurrent HF with reduced ejection fraction (HFrEF) and AF (HFrEF_AF) is still unclear. Furthermore, the role of SGLT2i in HFrEF_AF patients is also not fully understood. The purpose of this study is to compare the differences in EAT and GM between HFrEF_AF and HFrEF patients without AF (HFrEF_NAF), and to analyse the relationship between them and the patients' heart structure, as well as the changes of them after SGLT2i treatment.

**Methods:**

45 HFrEF_NAF patients and 39 HFrEF_AF patients were continuously recruited, with 26 patients from each group meeting the selection criteria. Fecal samples and CT scans were collected at baseline and after SGLT2i treatment for 3 months, respectively. The 16S rRNA gene of fecal samples was sequenced, and EAT volume (EATV) and EAT volume index (EATVI) values were measured and calculated with CT. These patients were followed up for up to 12 months to assess their long-term re-hospitalization rates.

**Results:**

The results showed that EATV [191.75 (119.59, 222.44) vs. 141.99 (97.10, 183.75)] and EATVI [105.07 (82.90, 116.39) mL/m^2^ vs. 81.03 (55.12, 108.74) mL/m^2^] in HFrEF_AF patients were significantly higher than in HFrEF_NAF patients. After adjusting for Age, Gender, CHD and BMI, Spearman correlation analysis showed that EATVI was significantly negatively correlated with post-conversion abundance of *Escherichia coli* (*R* = −0.496, *P* = 0.019) and *Escherichia Shigella* (*R* = −0.472, *P* = 0.027) in HFrEF_AF patients. The mediation analysis showed that *Escherichia Shigella* in HFrEF_AF patients had a significant indirect effect on LAAD through EATVI (ACME = −0.553, *P* = 0.042), but the direct and total effects were not significant. After 3 months of SGLT2i treatment, the connection between *Escherichia Shigella*, EATVI and LAAD disappeared in HFrEF_AF patients. Moreover, after SGLT2i treatment, harmful AF-related bacteria such as *Catenibacterium* are no longer enriched in HFrEF_AF patients, whereas beneficial bacteria such as *Blautia* are enriched. The 12 months follow-up showed no significant difference in re-hospitalization rate between HFrEF_NAF and HFrEF_AF patients.

**Conclusion:**

EATV and EATVI in HFrEF_AF patients were significantly higher than in HFrEF_NAF patients. There may be an abnormal “*Escherichia Shigella*-EAT-LAAD” axis in HFrEF_AF patients, and SGLT2i may benefit HFrEF_AF patients differently than HFrEF_NAF patients by inhibiting this axis.

**Clinical Trial Registration:**

https://www.chictr.org.cn/showproj.html?proj=240632, ChiCTR2400088128.

## Introduction

Heart failure with reduced ejection fraction (HFrEF) is characterized by typical heart failure symptoms and signs, as well as a left ventricular ejection fraction (LVEF) of ≤40%. It is usually associated with ventricular remodeling and decreased cardiac output. This is the most common type of heart failure (HF) (1, 2). In clinical practice, Atrial fibrillation (AF) and HFrEF frequently coexist, and they have complex pathophysiological interactions. AF can aggravate the progression of HF and significantly affects the prognosis of patients with HFrEF (3, 4).

Epicardial adipose tissue (EAT) is the visceral fat that covers the surface of the heart. It is closely related to myocardial metabolism, inflammation, and ventricular remodeling (5). Numerous recent studies have demonstrated that EAT is a significant factor in the onset and progression of a range of cardiovascular diseases, such as AF and HF (6, 7). Under pathological conditions, EAT can directly act on myocardial tissue to cause fibrosis by secreting various pro-fibrotic factors and pro-inflammatory cytokines. This could be an important mechanism for EAT to promote the development of AF (8).

Gut microbiota (GM) plays an important role in host-environment interaction, including immunity, metabolism, and cardiovascular regulation. GM imbalance has been associated to diseases such as diabetes, hypertension, and atherosclerosis. HFrEF patients have a reduced diversity of GM and a disorder in GM structure compared to healthy people, indicating a potential GM mechanism in HF (9). Recent studies discovered that transplanting GM from young animals into elderly rats can improve their intestinal barrier, inhibit NLRP3 inflammasome activity, and thus reduce the incidence of age-related AF (10). As a result, GM imbalance may play a significant role in the coexistence of HFrEF and AF. However, there has been little systematic research on the relationship between GM, EAT, and cardiac structure in HFrEF patients with AF.

Sodium-glucose co-transporter 2 inhibitor (SGLT2i), a class of novel glucose-lowering agents, have become one of the cornerstone drugs for the treatment of HFrEF (11). In addition to cardio-renal protective effects, SGLT2i may reverse ventricular remodeling and improve heart function in HFrEF patients by regulating GM composition, reducing EAT accumulation, improving myocardial mitochondrial function, anti-inflammation, and anti-fibrosis (12–16). Relevant studies have shown that SGLT2i can significantly reduce the incidence of AF and atrial flutter, but the mechanism is still unclear (17–19).

Therefore, this study enrolled patients with HFrEF as subjects: those without AF (HFrEF_NAF) were defined as the control group, and those with AF (HFrEF_AF) as the experimental group. The objectives of this study were to: First, Evaluate the differences in EAT and GM between the two groups. Second, Analyze the relationships of EAT and GM with cardiac structure and function in each group. Third, Explore the effect of SGLT2i on the associations between EAT, GM, and cardiac structure in both groups. Fourth, Investigate the re-hospitalisation rate within 12 months in both groups of patients.

## Materials and methods

### Sample collection

This prospective study included HFrEF_NAF and HFrEF_AF who visited the Qinghai Cardio-Cerebrovascular Specialty Hospital and the Affiliated Hospital of Qinghai University from August 2024 to October 2024. All patients who participated in this study had previously been hospitalized for HF. After receiving routine medical treatment (Beta-blockers, ACEI and MRA), they were readmitted to the hospital for additional treatment. Upon admission, HFrEF patients received anti-heart failure therapy with the addition of dapagliflozin (10 mg) to their baseline regimen. Dosages were adjusted based on clinical status, and medication adherence was monitored via telephone follow-up. HFrEF and persistent AF diagnosis meet the criteria established by the European Society of Cardiology (20, 21). All subjects were patients with New York Heart Association (NYHA) Functional Classification III–IV. To reduce confounding factors, patients who had received antibiotic therapy for any infection within 30 days prior to enrollment were excluded.

We collected 39 fecal samples from HFrEF_NAF patients, 32 fecal samples from HFrEF_AF patients, 45 computed tomography (CT) data from HFrEF_NAF patients, and 30 CT data from HFrEF_AF patients. However, there were only 26 HFrEF_NAF patients and 26 HFrEF_AF patients who had both fecal samples and CT data, for a total of 52 cases. The following demographic and clinical characteristics were also gathered and extracted: age, gender, body surface area (BSA), and BMI. Traditional cardiovascular risk factors include smoking, excessive alcohol consumption, high blood pressure, diabetes mellitus, and coronary heart disease (CHD). Key laboratory data, including triglycerides (TG), total cholesterol (TC), low-density lipoprotein cholesterol (LDL), high-density lipoprotein cholesterol (HDL), c-reactive protein (CRP), creatinine, fasting blood glucose (FBG) and uric acid (UA), were also collected. All biochemical measurements are performed in the hospital laboratory. All patients extracted the following data from echocardiography: LVEF, left ventricular end-systolic diameter (LVESD), left ventricular end-diastolic diameter (LVEDD), left atrial anterior-posterior diameter (LAAD) and right ventricular diameter (RVD).

### Ethics statement

This study has been approved by the Ethics Committee of the Qinghai Cardio-Cerebrovascular Specialty Hospital (No: QXYYLL-2024-93) and the Ethics Committee of the Affiliated Hospital of Qinghai University (No: P-SL-20230293), and the informed consent was signed by patients. This study has been registered in China Clinical Trial Registry (ChiCTR) (Trial registration number: ChiCTR2400088128).

### Acquisition and analysis of CT

All patients participating in this study underwent non-ECG-gated chest CT scans after enrollment and 3 months after adding SGLT2i. In this study, two CT scanners were used (uCT760 and uCT860, United Imaging, Shanghai, China). Scanning parameters include thin layer imaging (layer thickness: 1.0–1.5 mm), tube voltage of 120 kV, and automatic tube current adjustment (range: 10–286 mA). All data is anonymously processed, exported and stored in DICOM format. EAT segmentation was carried out using Mimics Medical 20.0 software and an inter-slice annotation approach, in which annotations were drawn every other slice to generate a three-dimensional reconstruction (22). The average EATV was computed using Mimics Medical 20.0 software (Materialise NV, https://www.materialise.com/en/healthcare), which allows for precise segmentation and 3D reconstruction of medical imaging data. Subsequently, EATVI was further calculated based on the patient's BSA. A medical student who had received specialise training performed the image segmentation manually, which was then reviewed and refined by an experienced radiologist. In addition, a seasoned cardiovascular specialist performed manual EAT segmentation to assess the reliability of the segmentation results. The inter-observer correlation coefficient (ICC) for EATV between the two segments was 0.900, indicating high inter-observer reliability.

### Fecal samples collection

All patients underwent fasting collection of fecal samples at baseline and 3 months after SGLT2i treatment, respectively. The collected fecal samples were frozen and stored in a −80 °C refrigerator within 2 h until further processing.

### DNA extraction and PCR amplification

The soil DNA Kit (DP336, TIANGEN, China) was used to extract total DNA, following the manufacturer's instructions. The DNA quality and concentration were detected using the Qubit@2.0 Fluorometer (Thermo Fisher Scientific, USA). By using the primers 515F (GTGCCAGCMGCCGCGGTAA) and 806R (GGACTACNNGGGTATCTAAT), the V3-V4 region of the bacterial 16S rRNA gene was amplified. PCR products were purified using the Universal DNA Purification and Recovery Kit (DP214-03, TIANGEN, CHINA) and quantified using the Qubit@2.0 Fluorometer (Thermo Fisher Scientific, USA), both according to the manufacturer's instructions. All samples were paired-end sequenced on the Illumina platform (insert size 300 bp, read length 125 bp) at the Novogene Bioinformatics Technology Co., Ltd. All methods were performed in accordance with the relevant guidelines and regulations. The PCR settings were as followings: thermal cycling consisted of initial denaturation at 98 °C for 1 min, followed by 30 cycles of denaturation at 98 °C for 10 s, annealing at 50 °C for 30 s, and elongation at 72 °C for 30 s and 72 °C for 5 min. All PCR reactions were carried out with 15 µL of Phusion High- Fidelity PCR Master Mix (DP336-02, New England Biolabs, USA), 0.2 µM of forward and reverse primers, and about 10 ng template DNA. All methods were performed in accordance with the relevant guidelines and regulations.

### Processing of sequencing data

After splicing each sample with Flash software (23), the raw data from paired-end sequencing were quality-filtered with Fastp software (Version 0.23.1) (24). The raw data were compared to the Silva database to identify chimeric sequences. The effective data were obtained by removing the chimeric sequences using the Vsearch Package (V2.16.0) (25). Using UPARSE, operational taxonomic units with a 97% similarity criterion were grouped (26). The taxonomy of each OTU representative sequence was assessed against the 16S rRNA database (Silva v138.1) using the Mothur Classifier version with an 80% confidence threshold (27).

### Clinical follow-up

We followed up with all patients for up to 12 months after adding SGLT2i treatment. All patients were followed up in the hospital's outpatient department, and their outcome status were determined using medical records and telephone interviews with patients and relatives. The number of hospital readmissions due to cardiovascular events among HFrEF patients will be recorded over a 12-month follow-up period. There were no patients lost during the follow-up.

### Statistical analysis

Statistical analysis was performed using SPSS25.0 statistical software. When the statistics of continuous variables follow a normal distribution, the results are expressed as means and standard deviations. When statistics for continuous variables do not conform to a normal distribution, the results are expressed as median and interquartile range. The differences between the two groups are analyzed using the the Student's *T* test (normal distribution data) or the Mann–Whitney *U* test (non-normal distribution data).

The alpha-diversity (including Observed-otus, Chao index, Shannon index, and Simpson index) of bacteria was calculated using the QIIME software (Version 1.9.1). Draw the rarefaction-curve with R software. The QIIME software (Version 1.9.1) was used to calculate beta diversity on both weighted and unweighted unifracs in order to assess differences in species complexity between samples. The LEfSe analysis (LDA score > 3.0) was performed to find the potential bacterial biomarkers of different groups.

For correlation and mediation analysis, we used Python (version 3.8) to perform a Centered Log-Ratio Transformation (CLR) on GM relative abundance data. SPSS (version 25.0) was used to perform correlation and Spearman partial correlation analyses. The R software (version 4.5.1) was used to conduct mediation analysis. Use the mediation package to determine the indirect effect of independent variables on dependent variables via mediating variables. The average causal mediation effects (ACME), average direct effect (ADE), and total effect were calculated using the Bootstrap method (5,000 replicates), and the effect was considered statistically significant if the 95% confidence interval did not include 0.

Patient survival curve analysis: Kaplan–Meier survival analysis was used to compare the time to re-hospitalization between the two groups, and statistical significance was determined using a log-rank test. For all data requiring a variance analysis, a *P*-value of less than 0.05 was considered statistically significant.

## Results

### Baseline characteristics of the study cohort

This study included 52 patients, including 26 HFrEF_NAF patients and 26 HFrEF_AF patients. The baseline clinical characteristics of the two groups were shown in Table 1. Other baseline clinical characteristics were not statistically different between the two groups, with the exception of concurrent CHD (18 vs. 8, *P* = 0.012). In terms of cardiac structure and function, there were statistical differences in LAAD [44.00 ± 6.00 vs. 50.00 (44.00, 57.00), *P* = 0.003] between the two groups, but no differences in other indicators.

**Table 1:** Baseline characteristics of the study population.

Variable	HFrEF_NAF (*n* = 26)	HFrEF_AF (*n* = 26)	*P* value
Age [years ± SD]	65.73 ± 10.30	65.57 ± 12.26	0.959
Male [*n*/*N* (%)]	15 (57.69)	16 (61.54)	1
BSA [m^2^ ± SD]	1.69 ± 0.14	1.79 ± 0.22	0.06
BMI [kg/m^2^ ± SD]	24.10 ± 4.17	24.90 ± 4.30	0.502
TG [mmol/L, M (P_25_, P_75_)]	1.23 (1.00, 1.72)	0.98 (0.81, 1.55)	0.083
TC [mmol/L, M (P_25_, P_75_)]	3.27 (2.49, 4.33)	3.34 (2.75, 4.16)	0.883
LDL [mmol/L, M (P_25_, P_75_)]	1.79 (1.29, 2.71)	1.97 (1.28, 2.67)	0.749
HDL [mmol/L ± SD]	0.88 ± 0.22	0.90 ± 0.23	0.742
CRP Abnormal [*n*/*N* (%)]	9 (34.62)	8 (30.77)	1
Creatinine [μmol/L, M (P_25_, P_75_)]	93.00 (73.75, 130.75)	94.85 (79.00, 118.50)	0.842
FBG [mmol/L, M (P_25_, P_75_)]	5.75 (4.40, 8.05)	5.30 (4.89, 6.36)	0.539
UA [μmol/L ± SD]	472.26 ± 176.81	505.61 ± 137.55	0.441
Smoking [*n*/*N* (%)]	7 (26.92)	7 (26.92)	1
Excessive alcohol consumption [*n*/*N* (%)]	4 (15.38)	5 (19.23)	1
Hypertension [*n*/*N* (%)]	15 (57.69)	14 (53.85)	1
Diabetes mellitus [*n*/*N* (%)]	10 (38.46)	9 (34.62)	1
CHD [*n*/*N* (%)]	18 (69.23)	8 (30.77)	0.012*
LVEF [%, M (P_25_, P_75_)]	38.00 (30.25, 40.00)	40.00 (34.75, 40.00)	0.263
LVESD [mm ± SD]	47.35 ± 10.29	47.54 ± 11.05	0.948
LVEDD [mm ± SD]	58.88 ± 9.68	59.64 ± 9.67	0.775
LAAD [mm, M (P_25_, P_75_)]	44.00 ± 6.00	50.00 (44.00, 57.00)	0.003*
RVD [mm, M (P_25_, P_75_)]	24.50 (19.75, 28.25)	21.50 (19.00, 28.75)	0.615

BSA, body surface area; BMI, body mass index; TG, triglycerides; TC, total cholesterol; LDL, low-density lipoprotein cholesterol; HDL, high-density lipoprotein cholesterol; CRP, c-reactive protein; FBG, fasting blood glucose; UA, uric acid; CHD, coronary heart disease; LVEF, left ventricular ejection fraction; LVESD, left ventricular end-systolic diameter; LVEDD, left ventricular end-diastolic diameter; LAAD, left atrial anterior-posterior diameter; RVD, right ventricular diameter.

* *P* < 0.05

The changes in key laboratory data and cardiac ultrasound parameters between the two patient groups before and after SGLT2i treatment were further compared. The results showed that patients with HFrEF_NAF had a significant increase in LVEF [38.00 (30.25, 40.00) vs. 39.00 (33.50, 48.25), *P* = 0.015] and a decrease in TG levels [1.23 (1.00, 1.72) vs. 1.11 (0.84, 1.58), *P* = 0.035] after SGLT2i treatment. After treatment with SGLT2i, TC [3.34 (2.75, 4.16) vs. 2.88 (2.45, 3.72), *P* = 0.043] and LVEF [40.00 (34.75, 40.00) vs. 38.50 (32.75, 51.25), *P* = 0.034] were significantly reduced in HFrEF_AF patients (Table 2).

**Table 2:** Baseline characteristics of HFrEF_NAF patients and HFrEF_AF patients before and after treatment.

Variable	HFrEF_NAF (*n* = 26)			HFrEF_AF (*n* = 26)		
	Before	After	*P* value	Before	After	*P* value
TG [mmol/L, M (P_25_, P_75_)]	1.23 (1.00, 1.72)	1.11 (0.84, 1.58)	0.035*	0.98 (0.81, 1.55)	1.01 (0.86, 1.35)	0.083
TC [mmol/L, M (P_25_, P_75_)]	3.27 (2.49, 4.33)	2.92 (2.45, 3.77)	0.576	3.34 (2.75, 4.16)	2.88 (2.45, 3.72)	0.043*
LDL [mmol/L, M (P_25_, P_75_)]	1.79 (1.29, 2.71)	1.63 (1.33, 2.29)	0.726	1.97 (1.28, 2.67)	1.86 ± 0.71	0.134
HDL (mmol/L ± SD)	0.88 ± 0.22	0.89 ± 0.19	0.737	0.90 ± 0.23	0.85 ± 0.25	0.171
Creatinine [μmol/L, M (P_25_, P_75_)]	93.00 (73.75, 130.75)	90.50 (72.50, 111.25)	0.518	94.85 (79.00, 118.50)	98.50 (81.70, 126.00)	0.298
FBG [mmol/L, M (P_25_, P_75_)]	5.75 (4.40, 8.05)	5.85 (4.48, 7.55)	0.799	5.30 (4.89, 6.36)	5.34 (4.59, 6.25)	0.943
UA [μmol/L, M (P_25_, P_75_)]	472.26 ± 176.81	416.00 (337.25, 545.50)	0.159	505.61 ± 137.55	515.69 ± 182.93	0.605
LVEF [%, M (P_25_, P_75_)]	38.00 (30.25, 40.00)	39.00 (33.50, 48.25)	0.015*	40.00 (34.75, 40.00)	38.50 (32.75, 51.25)	0.034*
LVESD [mm ± SD]	47.35 ± 10.29	47.58 ± 9.51	0.884	47.54 ± 11.05	47.58 ± 9.10	0.677
LVEDD [mm ± SD]	58.88 ± 9.68	59.77 ± 8.75	0.541	59.64 ± 9.67	58.96 ± 6.70	0.958
LAAD [mm ± SD)	44.00 ± 6.00	43.69 ± 7.28	0.821	50.00 (44.00, 57.00)	50.00 (41.50, 59.25)	0.668
RVD [mm, M (P_25_, P_75_)]	24.50 (19.75, 28.25)	23.00 (20.00, 25.50)	0.613	21.50 (19.00, 28.75)	23.00 (17.75, 28.00)	0.234

TG, triglycerides; TC, total cholesterol; LDL, low-density lipoprotein cholesterol; HDL, high-density lipoprotein cholesterol; FBG, fasting blood glucose; UA, uric acid; LVEF, left ventricular ejection fraction; LVESD, left ventricular end-systolic diameter; LVEDD, left ventricular end-diastolic diameter; LAAD, left atrial anterior-posterior diameter; RVD, right ventricular diameter.

_*_P < 0.05

### The differences of EATV and EATVI between HFrEF_NAF and HFrEF_AF patients at baseline and after SGLT2i treatment

**Figure 1:**
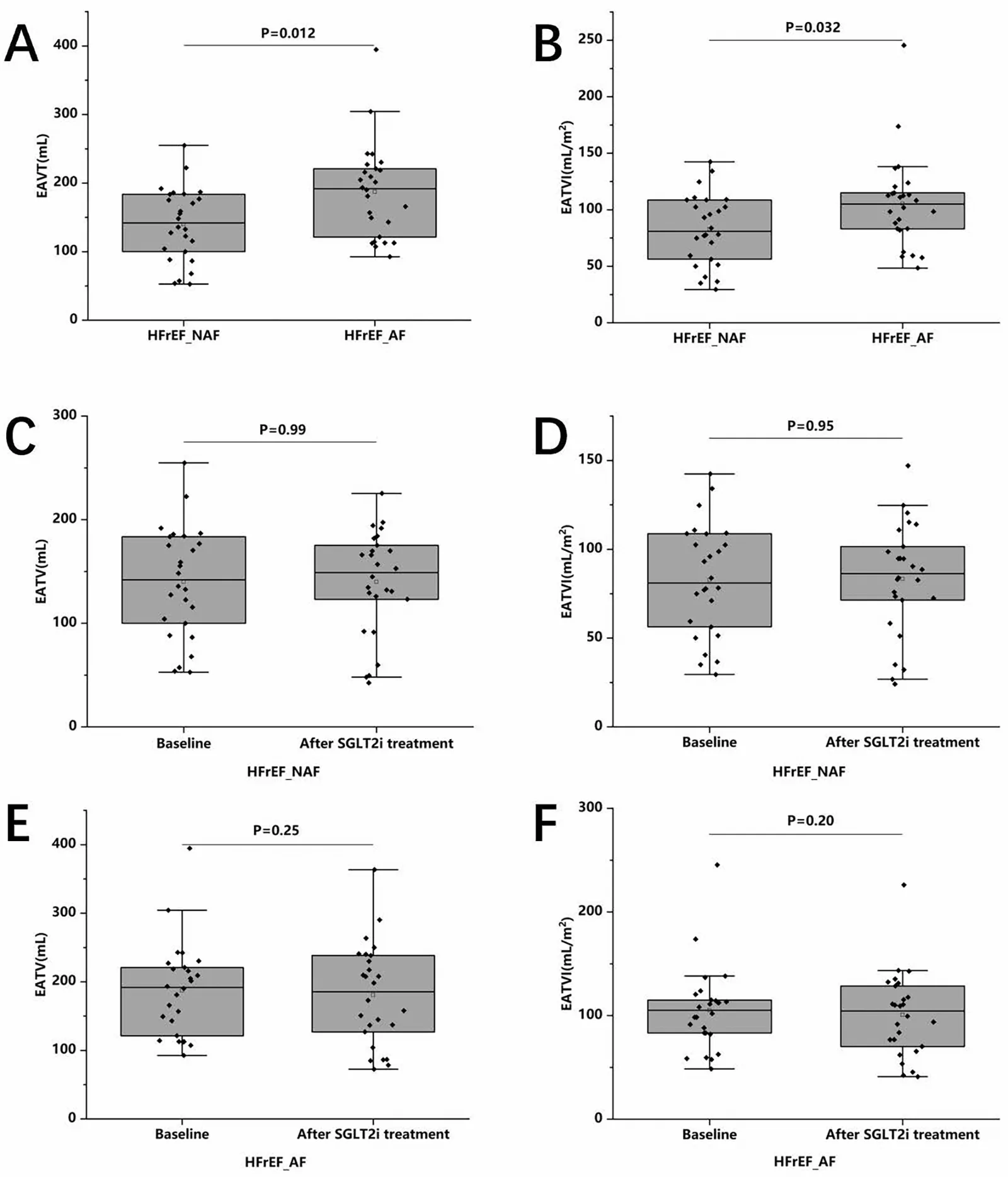
Comparison of EATV and EATVI between HFrEF_NAF patients and HFrEF_AF patients before and after SGLT2i treatment. The differences of EATV and EATVI between HFrEF_NAF patients and HFrEF_AF patients are shown in **(A)** and **(B)**, respectively. The differences of EATV and EATVI between before and after SGLT2i treatment in HFrEF_NAF patients are shown in **(C)** and **(D)**, respectively. The differences of EATV and EATVI between before and after SGLT2i treatment in HFrEF_AF patients are shown in **(E)** and **(F)**, respectively.

CT imaging results showed that EATV in HFrEF_NAF patients was 141.99 (97.10, 183.75) mL, while it was 191.75 (119.59, 222.44) mL in HFrEF_AF patients. EATV was significantly higher in HFrEF_AF patients compared to HFrEF_NAF patients, and the difference was statistically significant (*P* = 0.012) (Figure 1A). EATVI in HFrEF_NAF patients was 81.03 (55.12, 108.74) mL/m^2^, while it was 105.07 (82.90, 116.39) mL/m^2^ in HFrEF_AF patients. EATVI in HFrEF_AF patients was significantly higher than that in HFrEF_NAF patients, and the difference was statistically significant (*P* = 0.032) (Figure 1B). After 3 months of SGLT2i treatment, there were no significant changes in EATV and EATVI in HFrEF_NAF Patients (Figures 1C,D). Although EATV and EATVI decreased in HFrEF_AF patients, there were no statistical differences (Figures 1E,F).

### The changes of GM in HFrEF_NAF and HFrEF_AF patients at baseline and after SGLT2i treatment

A total of 104 fecal samples were successfully sequenced, and a total of 2,237 OTUs were obtained from all samples (Supplementary Figure S1A). The rarefaction curve shows a plateau period of species richness, indicating that the sequencing depth in this study is sufficient (Supplementary Figure S1B). At the same time, we drew a box plot of species accumulation, and the results showed that the majority of the microbial diversity had been captured, indicating that the number of samples and species richness in this study were adequate for in-depth discussion and analysis (Supplementary Figure S1C).

The Alpha diversity index was calculated to assess the richness and diversity of GM. The results showed that there were no significant differences in the diversity and richness of GM between HFrEF_NAF and HFrEF_AF patients. After SGLT2i treatment, the diversity and richness of GM increased in both HFrEF_NAF and HFrEF_AF patients, but there was no statistical difference (Supplementary Figure S2). At the same time, we calculated Beta Diversity through NMDS to assess the differences in GM between HFrEF_NAF patients and HFrEF_AF patients. The results showed that there were a few variations in GM between HFrEF_NAF patients and HFrEF_AF patients before and after SGLT2i treatment, but there was no statistical difference (Supplementary Figure S3).

**Figure 2:**
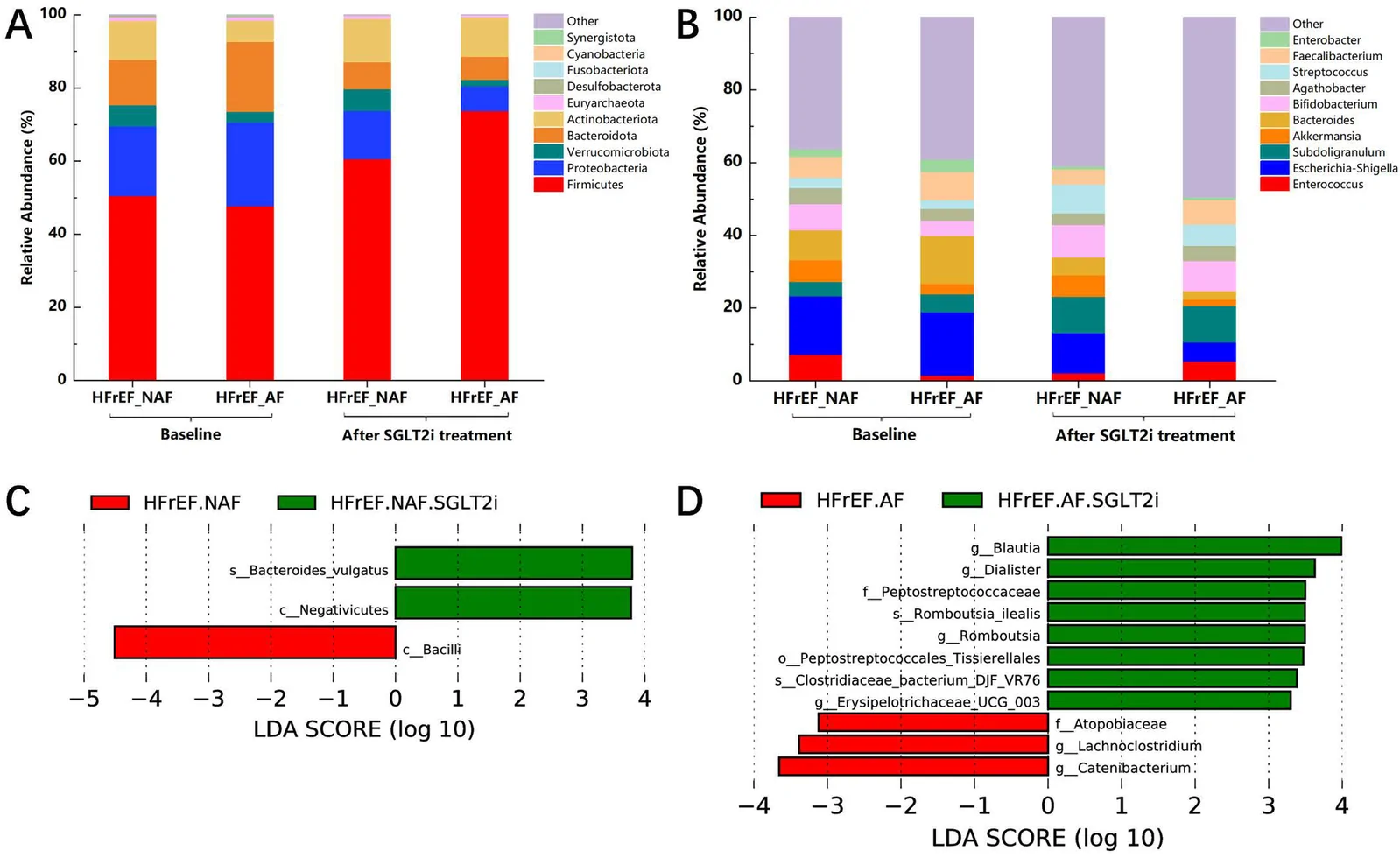
Abundance distribution of GM in HFrEF_NAF patients and HFrEF_AF patients at baseline and after SGLT2i treatment, respectively. This bar charts show the relative abundance of bacterial communities at the phylum **(A)** and genus **(B)** levels in HFrEF_NAF patients and HFrEF_AF patients at baseline as well as after SGLT2i treatment, respectively. The LEfSe plot shows the respective dominant bacteria in HFrEF_NAF patients **(C)** and HFrEF_AF patients **(D)** before and after treatment with SGLT2i.

To evaluate the difference in GM distribution between HFrEF_NAF patients and HFrEF_AF patients before and after treatment, we created bar graphs showing the relative abundance of their top 10 GM at the plylum and genus levels (Figures 2A,B). To investigate the differential abundance of individual taxa, we performed a statistical analysis of the relative abundance of each taxon in HFrEF_NAF and HFrEF_AF patients at various phylogenetic ranks. After SGLT2i treatment, the levels of Erysipelotrichales and Actinomycetales decreased at the phylum level in HFrEF_NAF patients; at the family level, the level of Veillonellaceae increased while the level of Actinomycetaceae decreased in HFrEF_NAF patients; and at the genus level, the level of *Actinomyces* decreased (Supplementary Figure S4). There were significant differences in one phylum, two orders, three families, six genera and two species in HFrEF_AF patients before and after treatment (Supplementary Figure S5). After treatment with SGLT2i, the levels of Firmicutes increased at the phylum level in HFrEF_AF patients; the levels of Veillonellales-Selenomonadales and Clostridia_UCG-014 increased at the order level in HFrEF_AF patients; the levels of Veillonellaceae, Peptostreptococcaceae, and Butyriciciccaceae increased at the family level in HFrEF_AF patients; the levels of *Blautia*, *Erysipelotrichaceae_UCG-003*, *Romboutsia*, *Ruminococcus_gauvreauii_group*, and *Butyricicoccus* increased at the genus level, while the level of *Slackia* decreased in HFrEF_AF patients; the level of *Romboutsia_ilealis* increased at the species level, while the level of *Slackia_isoflavoniconvertens* decreased in HFrEF_AF patients.

The LEfSe analysis was performed to find the potential bacterial biomarkers related to different groups of patients. At the genus level, *Catenibacterium* and *Lachnoclostridium* were enriched in pre-treatment HFrEF_AF patients (LDA = 3.700, *P* = 0.037; LDA = 3.385, *P* = 0.039, respectively). After 3 months of SGLT2i treatment, *Blautia*, *Dialister*, *Romboutsia* and *Erysipelotrichacea*_*UCG_003* were significantly enriched in HFrEF_AF patients (LDA = 3.991, *P* = 0.005; LDA = 3.637, *P* = 0.049; LDA = 3.464, *P* = 0.017; LDA = 3.347, *P* = 0.029, respectively) (Figures 2C,D).

The functional gene composition of GM was analyzed by the PICRUSt to differentiate the normal biological function between HFrEF_NAF patients and HFrEF_AF patients. For KEGG level 1, there was no difference between HFrEF_NAF patients and HFrEF_AF patients before and after treatment. For KEGG level 2, the metabolism and glycan biosynthesis and metabolism were significantly higher in HFrEF_AF patients before SGLT2i treatment (Supplementary Figure S6). For KEGG level 3, glycolysis/gluconeogenesis and selenocompound metabolism were significantly higher in HFrEF_NAF patients before SGLT2i treatment, while energy metabolism was much higher in HFrEF_NAF patients after SGLT2i treatment. secretion system, bacterial secretion system, citrate cycle, membrane and intracellular structural molecules, lipopolysaccharide biosynthesis proteins, pores ion channels, lipopolysaccharide biosynthesis, ubiquinone and other terpenoid-quinone biosynthesis, lysine degradation and aminobenzoate degradation were significantly higher in HFrEF_AF patients before SGLT2i treatment, while chromosome, lysine biosynthesis, peptidoglycan biosynthesis, mismatch repair, cell cycle-caulobacter and photosynthesis were much higher in HFrEF_AF patients after SGLT2i treatment (Supplementary Figure S7).

### The relationships between GM, EATVI, and LAAD in HFrEF_NAF and HFrEF_AF patients

**Figure 3:**
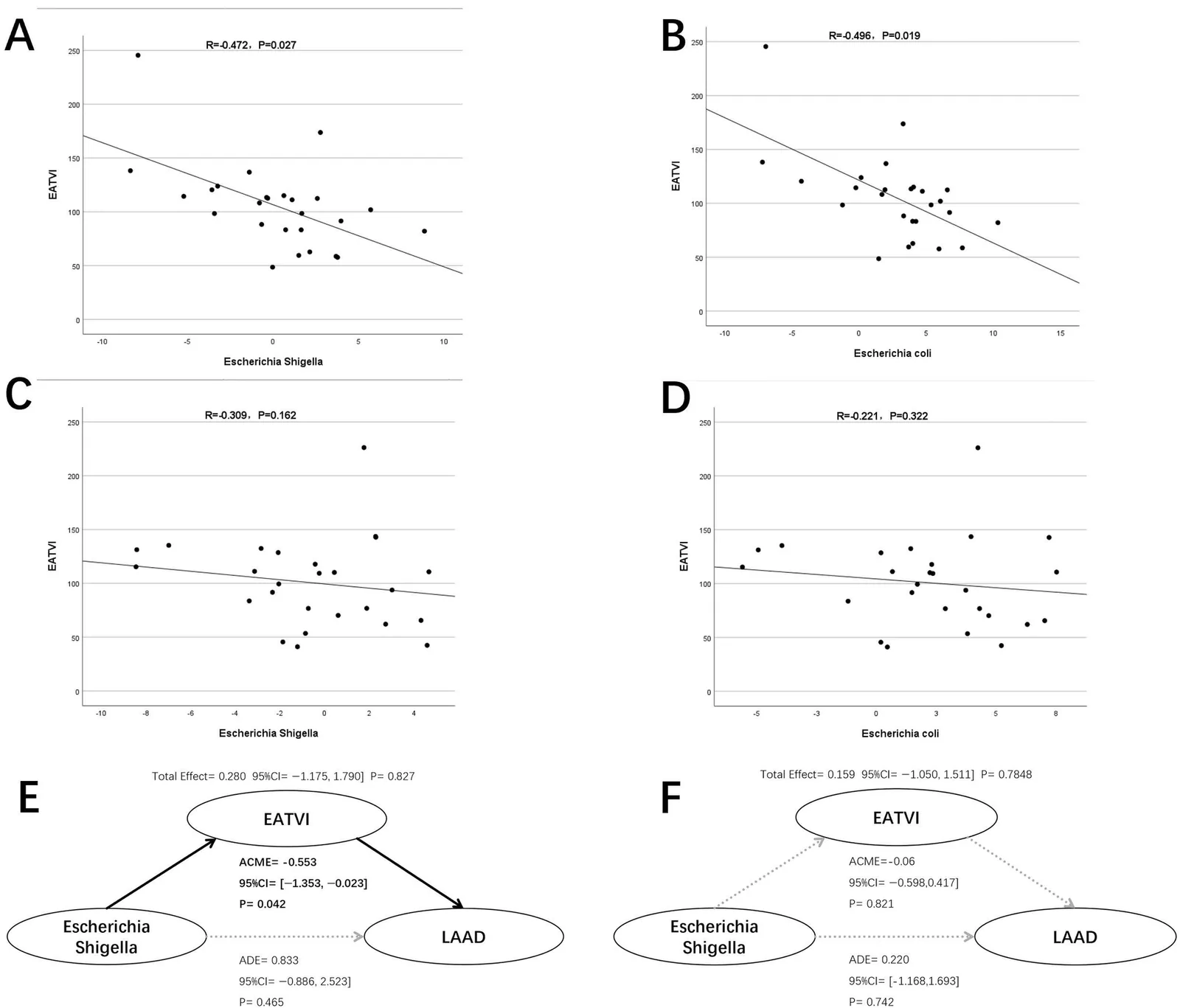
Correlation analysis and mediation analysis between GM, EATVI and LAAD. The linear correlations of *Escherichia Shigella* and *Escherichia coli* with EATVI at baseline level are shown in **(A)** and **(B)**, respectively. The linear correlations of *Escherichia Shigella* and *Escherichia coli* with EATVI after SGLT2i treatment are shown in **(C)** and **(D)**, respectively. Mediation analysis between *Escherichia Shigella*, EATVI and LAAD in HFrEF_AF patients at baseline level and after SGLT2i treatment are shown in **(E)** and **(F)**, respectively.

Following CLR conversion of various levels of GM in HFrEF_NAF and HFrEF_AF patients, Spearman correlation analysis was used to determine the relationship between GM and EATVI. The results showed that, whether before or after SGLT2i treatment, the post-conversion abundance of GM at all levels in HFrEF_NAF patients did not correlate with their EATVI. In HFrEF_AF patients, the post-conversion abundance of Actinobacterota at the phylum level showed a positive correlation with EATVI (*R* = 0.406, *P* = 0.039), while Proteobacteria showed a negative correlation with EATVI (*R* = −0.396, *P* = 0.045). At the order level, Veillonellales_Selenomonadales showed a negative correlation with EATVI (*R* = −0.441, *P* = 0.024). At the genus level, *Enterococcus* was positively correlated with EATVI, while *Escherichia Shigella* was negatively correlated (*R* = 0.437, *P* = 0.025; *R* = −0.569, *P* = 0.002, respectively). At the special level, *Escherichia coli* and *Enterobacter hormaechei* both showed a negative correlation with EATVI (*R* = −0.534, *P* = 0.005; *R* = −0.398, *P* = 0.044, respectively) (Supplementary Figures S8A–G). After adjusting for Age, Gender, CHD and BMI, Spearman's partial correlation analysis showed that the post-conversion abundance of *Escherichia Shigella* and *Escherichia coli* was significantly negative correlated with EATVI (*R* = −0.472, *P* = 0.027; *R* = −0.496, *P* = 0.019, respectively) (Figures 3A,B). However, after 3 months of treatment with SGLT2i, the post-conversion abundance of *Escherichia Shigella* and *Escherichia coli* was no longer correlated with EATVI in HFrEF_AF patients (*R* = −0.309, *P* = 0.162; *R* = −0.221, *P* = 0.322, respectively) (Figures 3C,D). The correlation between EATVI and LAAD was only marginally significant (*R* = 0.349, *P* = 0.081) (Supplementary Figure S9). Combined with previous studies, the correlation between EATVI and LAAD has been clearly confirmed, which is considered to be related to the small sample size of this study.

Furthermore, we used mediation analyses to determine whether *Escherichia Shigella* and *Escherichia coli* regulate LAAD in HFrEF_AF patients through EAT. The results showed that while *Escherichia Shigella* had no significant total effect and direct effect on LAAD, it did have a significant indirect effect on LAAD through EATVI [ACME  = −0.553, 95% CI (−1.353, −0.023), *P* = 0.042; ADE = 0.833, 95% CI (−0.886, 2.523), *P* = 0.465; Total Effect = 0.280, 95% CI (−1.175, 1.790), *P* = 0.827] (Figure 3E). However, after 3 months of SGLT2i treatment, *Escherichia Shigella* no longer had an indirect effect on LAAD through EATVI (ACME = −0.06, 95% CI [−0.598, 0.417], *P* = 0.821) (Figure 3F). However, neither at the baseline level nor after 3 months of SGLT2i treatment did *Escherichia coli* exert an effect on LAAD through EATVI (data are not shown).

### Analysis of long-term re-hospitalization rates between HFrEF_NAF patients and HFrEF_AF patients

**Figure 4:**
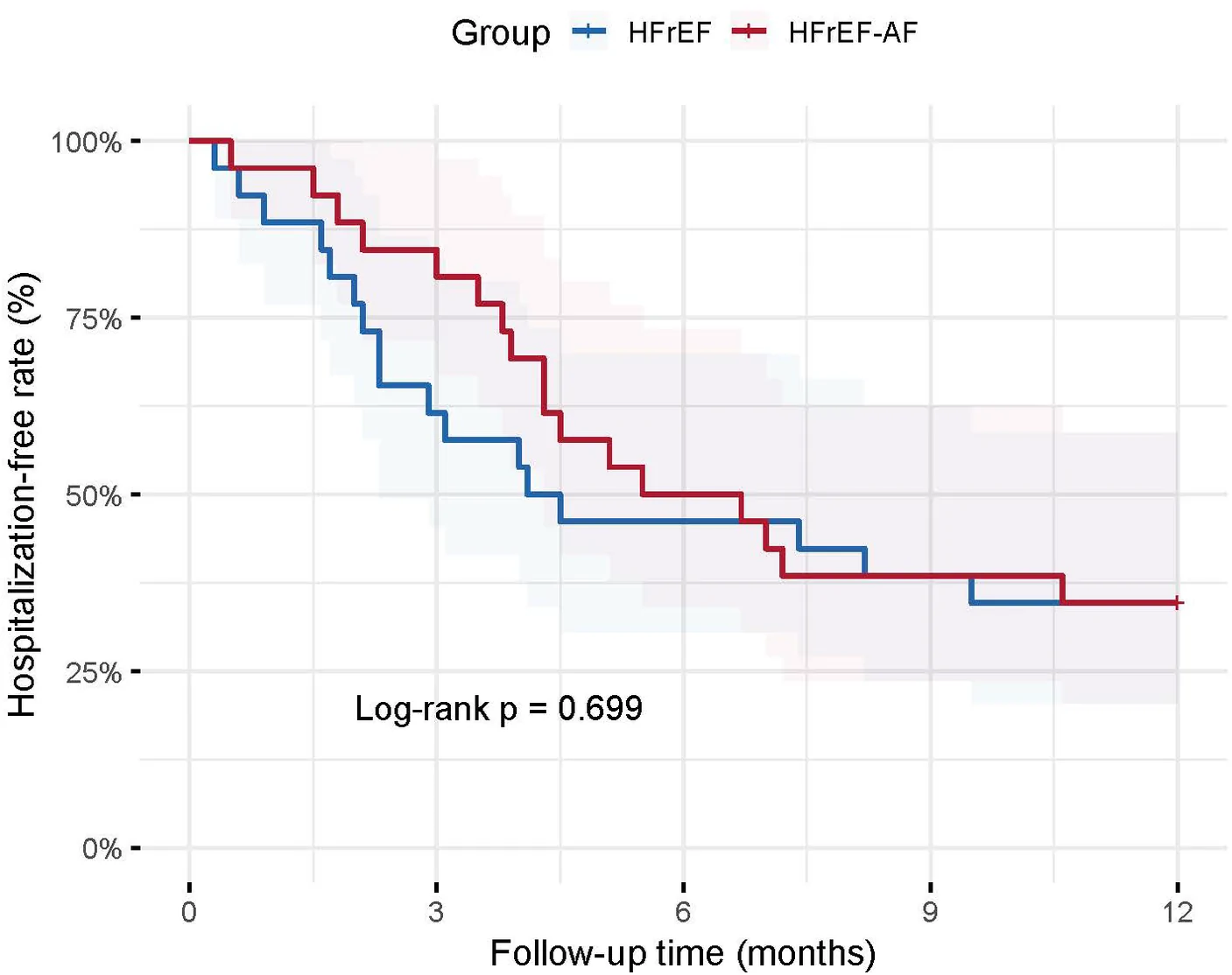
Analysis of long-term hospitalization rates between HFrEF_NAF patients and HFrEF_AF patients.

During the 12 months follow-up period, Kaplan–Meier analysis showed no significant differences in re-hospitalization rates between the HFrEF_NAF and HFrEF_AF patients (*P* = 0.699) (Figure 4). It indicates that SGLT2i have a similar effect on reducing the risk of long-term hospitalization in HFrEF_AF patients as in HFrEF_NAF patients.

## Discussion

LAAD represents the size of the left atrium and is the most important indicator for predicting the risk of AF and assessing the prognosis of AF patients (28). In HFrEF patients, LAAD has been shown to be an important independent risk factor for predicting their risk of AF, thrombotic events, and all-cause death (29). In this study, we first confirmed that LAAD was significantly higher in HFrEF_AF patients than in HFrEF_NAF patients. However, the pathological factors contributing to changes in LAAD remain incompletely understood.

As EAT is in direct contact with the heart and is supplied by the coronary arteries, it can exert a direct effect on the heart via endocrine and paracrine pathways. Under physiological conditions, EAT secretes bioactive molecules that maintain the balance of anti-inflammatory and pro-inflammatory molecules in the myocardium while also protecting the heart's structure and function (30). However, EAT is also involved in the pathological changes of cardiovascular disease. In recent years, numerous studies have verified that when EAT structure and function alter as a result of different factors, dysfunctional EAT can promote myocardial cell hypertrophy and apoptosis by inducing inflammatory reactions, lipid infiltration, and oxidative stress, resulting in myocardial fibrosis, changes in cardiac structure and electrical remodelling (31, 32). On the one hand, excessive elevation of EAT leads to overexpression of inflammatory mediators, and oxidative stress as well as secretion of inflammatory cytokines are closely associated with EAT volume. EAT-derived IL-6 has the ability to increase inflammatory cell infiltration, cause cardiac fibrosis, activate the NF-κB pathway, and increase the duration of atrial muscle cell action potentials (33). The increase in leptin from EAT mediates aldosterone-induced oxidative stress in endothelial cells, increased myocardial fibrosis, vascular dysfunction, and enhanced inflammation, eventually leading to cardiac remodeling (34). On the other hand, an excessively elevated EAT will mechanically occupy and compress the myocardium, resulting in problems with cardiac filling, and then slowly infiltrate the myocardium to alter its ultrastructure, which will alter the structure and function of the myocardium. EAT produces free fatty acids, which lead to abnormal myocardial lipids and myocardial cell disorders (35). These factors work together to alter the structure and function of the heart, which raises the risk of HF and AF significantly. Recent studies have confirmed that EATV is independently associated with the risk of new AF in the population, with higher EATV increasing the risk of developing AF (36). Increased EAT causes atrial structural remodeling and fibrosis by expressing pro-fibrotic factors, and plays a role in the occurrence and maintenance of AF (37). However, studies by Jin et al. have shown that EAT is significantly reduced in patients with HFrEF, which is attributed to the state of physical exhaustion in these patients (38). The results of this study showed that EATV and EATVI in HFrEF_AF patients were significantly higher than in HFrEF_NAF patients, confirming that patients with AF had a significantly higher amount of EAT than those without AF. Previous studies have confirmed the link between EAT and LAAD. According to Nabati and Çiçek's research, there is a correlation between EAT thickness and LAAD (39, 40). However, the correlation between EATVI and LAAD exhibits a marginal trend in our study, which is considered to be due to the small sample size. Although the studies mentioned above suggest that EAT may play a role in the development and maintenance of AF in HFrEF patients, the regulatory factors that precede EAT remain unknown.

GM has an important role in human physiology and pathology. They can prevent endotoxin from invading the body's internal circulation and causing disease by maintaining host intestine homeostasis (41). The imbalance of GM has been proved to be related to the occurrence and development of various cardiovascular diseases, including CHD, HF and arrhythmia (42). The relationship between GM and HF and AF has been widely confirmed (43). At the same time, GM plays a role in the metabolism of various nutrients in the body, including sugars and lipids. An imbalance in the structure of GM can cause changes in the body's energy and fat storage, including lipid metabolism disorders, abnormalities in blood lipid indicators such as fatty acids, TG, and TC, resulting in abnormal fat tissue accumulation and metabolism (44, 45). Based on the preceding research, we analyzed the relationship between the abundance of various levels of GM and EATVI in order to clarify the correlation between EATVI and related bacterial groups. The results of this study showed that after adjusting for possible confounding factors such as age, gender, and BMI, *Escherichia Shigella* and *Escherichia coli* were still significantly negatively correlated with EATVI in HFrEF_AF patients, but not in HFrEF_NAF. Preliminary findings indicate a possible connection between *Escherichia Shigella*, *Escherichia coli*, and EAT in HFrEF_AF patients. Based on the correlation between *Escherichia Shigella*, *Escherichia coli*, and EATVI, as well as the correlation between EATVI and LAAD in HFrEF_AF patients, we used mediation analysis to determine whether *Escherichia Shigella* and *Escherichia coli* affect the prognosis of HFrEF_AF patients through EAT. The results confirmed that *Escherichia Shigella* had a significant indirect effect on LAAD through EATVI in HFrEF_AF patients, but no significant direct effect was observed. Therefore, we speculate that there is a special “*Escherichia Shigella*-EAT-LAAD” axis in HFrEF_AF patient. *Escherichia Shigella* may affect the size of LAAD by inhibiting the volume of EAT, and EAT may play an important role in *Escherichia Shigella*'s impact on AF.

Currently, SGLT2i has been widely confirmed to benefit HF patients by improving mitochondrial function, reducing oxidative stress, and providing anti-fibrotic effects, and SGLT2i can reduce the risk of new AF (46–48). However, it is unclear whether the presence of AF affects SGLT2i's cardiovascular efficacy in patients with chronic HF. As a result, this study expanded on the efficacy of SGLT2i in HFrEF_AF patients, as well as the mechanism involved. The results of this study showed that HFrEF_AF patients had an abnormal “*Escherichia Shigella*-EAT-LAAD” axis, which was severed 3 months after adding SGLT2i. There is no evidence that SGLT2i can directly affect *Escherichia Shigella*, but related research has shown that SGLT2i can benefit patients by regulating EAT. Although the effect of SGLT2i on the EAT of these patients in this study was not as large as expected due to the short duration of administration of SGLT2i, the abnormal “*Escherichia Shigella*-EAT-LAAD” axis in patients with HFrEF_AF was indeed cut. However, the point of action of SGLT2i is still unknown, so additional animal experiments may be required to confirm the specific mechanism. Moreover, our results showed significant differences in the GM structure between HFrEF_AF patients before and after SGLT2i treatment. The results showed that at the genus level of GM, HFrEF_AF patients were enriched in *Catenibacterium* before SGLT2i treatment, while HFrEF_AF patients were enriched in *Blautia* after SGLT2i treatment, and the increase or decrease of these two bacteria may have a negative impact on human health. The causative relationship between the increase in *Catenibacterium* and the occurrence of AF has been proven (49). Recent studies have found a significant increase in *Catenibacterium* in people with CAD in mid-to-high altitude areas, which may be related to their unique dietary characteristics, namely higher red meat intake (50). Consuming red meat increases Trimethylamine N-oxide (TMAO) production in the GM metabolite. High circulating TMAO concentrations have been associated to an increased risk of cardiovascular disease (51), while *Catenibacterium* is more abundant in people with high TMAO concentrations (52). The population in this study is primarily concentrated in mid-altitude areas. Our study discovered that *Catenibacterium* is more abundant in patients with HFrEF_AF, which may be related to the development of AF. *Blautia* affects the body in a variety of ways, including altering energy metabolism and decreasing inflammation (53–55). *Blautia* has been discovered to be inversely associated with visceral fat area, which is thought to be an obesity biomarker for cardiovascular and metabolic disease risk. *Blautia* can influence fatty acid metabolism, reduce the accumulation of harmful lipids in the liver, and change the composition of circulating bile acids that aid fat absorption (56, 57). In addition, *Blautia* has anti-inflammatory properties and is involved in the metabolism of carbohydrates, contributing to the increase of short-chain fatty acids (SCFAs), metabolic products of the GM (58, 59). SCFAs, one of the most important metabolites of the GM, has been proven to inhibit the synthesis and secretion of proinflammatory factors, reduce the body's inflammatory response, inhibit lipid formation, and improve glucose and lipid metabolism (60). Butyric acid, one of SCFAs, can stimulate the maturation of T cells on the surface of the intestinal mucosa, contribute to chronic immune activation, and prevent ventricular remodelling (61). Propionic acid, another one of SCFAs, has also been proven to contribute to maintain immune homeostasis by regulating T cell activation, which improves myocardial hypertrophy and fibrosis (62). Therefore, we concluded that after SGLT2i treatment, patients with HFrEF_AF no longer enrich *Catenibacterium*, but rather *Blautia*, which could be one of the reasons they can benefit from SGLT2i. Furthermore, we also observed significant differences in the function of GM in fecal samples from HFrEF_NAF patients and HFrEF_AF patients, which will influence GM colonisation and reproduction.

In addition, EATV and EATVI decreased after treatment in HFrEF_AF patients, though there was no statistical difference between them, which we attributed to the shorter treatment time with SGLT2i drugs. The LVEF decreased in HFrEF_AF patients after SGLT2i use, which may be due to bias caused by a small sample size. Recent research has shown that AF can significantly increase the risk of readmission, all-cause death, and cardiovascular death in HFrEF patients (63, 64). Our results show that, while EF values in HFrEF_AF patients decreased after SGLT2i treatment, there was no significant difference in re-hospitalization events within a year between HFrEF_NAF patients and HFrEF_AF patients, implying that SGLT2i may provide additional benefits to HFrEF_AF patients compared to HFrEF_NAF patients.

### Limitations

Although our study reveals the relationship between GM, EAT and cardiac functional structure in HFrEF_AF patients. However, it has several limitations. First, due to the difficulties in sample collection, the sample size in this study was relatively small. Therefore, this study serves as a preliminary exploration of the relationship among gut microbiota, epicardial adipose tissue, and SGLT2 inhibitors. Further validation with larger sample sizes is warranted. Second, AF is basically classified into two types: paroxysmal and persistent AF. To reduce heterogeneity among samples, this study primarily included HFrEF who had persistent AF. Third, a significant difference in the prevalence of coronary heart disease was observed between the HFrEF_AF and HFrEF_NAF patients. This disparity may exert independent effects on epicardial adipose tissue and gut microbiota. Therefore, future studies with a larger sample size are warranted to minimize this potential bias on the results. Fourth, changes in the gut microbiota cannot be ruled out as a consequence of clinical improvement following an acute heart failure episode. Fifth, this study did not adjust for all potential multivariate confounding factors. Sixth, although we excluded patients who had received antibiotic therapy for any infection within 30 days prior to enrollment were excluded, the medications taken by some diabetic patients may still influence the gut microbiota.

## Conclusions

The EATV and EATVI in HFrEF_AF patients were significantly higher than in HFrEF_NAF patients. There may be an abnormal “*Escherichia Shigella*-EAT-LAAD” axis in HFrEF_AF patients, and SGLT2i may benefit HFrEF_AF patients differently than HFrEF_NAF patients by inhibiting this axis. In HFrEF_AF patients, SGLT2i has a similar effect on reducing the risk of long-term re-hospitalization as in HFrEF_NAF patients. Given that AF can worsen the progression of HFrEF, our research results suggest that SGLT2i may provide additional benefits to HFrEF_AF patients.

## Data Availability

The datasets presented in this study can be found in online repositories. The names of the repository/repositories and accession number(s) can be found in the article/Supplementary Material.

## Supplementary material

Supplementary Figure S1 The richness of gut microbiota between HFrEF_NAF patients and HFrEF_AF patients.

Supplementary Figure S2 The alpha diversity indices of gut microbiota in all groups.

Supplementary Figure S3 The beta diversity of all groups.

Supplementary Figure S4 Gut microbiota differences in HFrEF_NAF patients before and after SGLT2i treatment at order **(A)**, family **(B)** and genus **(C)** levels.

Supplementary Figure S5 Gut microbiota differences in HFrEF_NAF patients before and after SGLT2i treatment at phylum **(A)**, order **(B)**, family **(C)**, genus **(D)**, and species **(E)** levels.

Supplementary Figure S6 Functional capability analysis based on the KEGG pathways (HFrEF_NAF patients before and after SGLT2i treatment).

Supplementary Figure S7 Functional capability analysis based on the KEGG pathways (HFrEF_AF patients before and after SGLT2i treatment).

Supplementary Figure S8 Correlation analysis between GM and EATVI at different levels.

Supplementary Figure S9 Correlation analysis between LAAD and EATVI.
